# Gut microbiota profiling in injection drug users with and without HIV-1 infection in Puerto Rico

**DOI:** 10.3389/fmicb.2024.1470037

**Published:** 2024-11-26

**Authors:** Nirosh Aluthge, Seidu Adams, Carmen A. Davila, Nova R. Gocchi Carrasco, Kathy S. Chiou, Roberto Abadie, Sydney J. Bennett, Kirk Dombrowski, Angel M. Major, Aníbal Valentín-Acevedo, John T. West, Charles Wood, Samodha C. Fernando

**Affiliations:** ^1^Department of Animal Science, University of Nebraska-Lincoln, Lincoln, NE, United States; ^2^Department of Sociology, University of Nebraska-Lincoln, Lincoln, NE, United States; ^3^Department of Psychology, University of Nebraska-Lincoln, Lincoln, NE, United States; ^4^Department of Kinesiology, University of Wisconsin-Madison, Madison, WI, United States; ^5^Department of Biological Science, University of Nebraska-Lincoln, Lincoln, NE, United States; ^6^University of Vermont, Burlington, VT, United States; ^7^Department of Microbiology and Immunology, Universidad Central del Caribe, Bayamon, Puerto Rico; ^8^Department of Interdisciplinary Oncology, Louisiana State University Health Sciences Center, Louisiana Cancer Research Center, New Orleans, LA, United States

**Keywords:** microbiome, 16S rDNA, HIV, drug use, bacterial community composition

## Abstract

**Introduction:**

The full extent of interactions between human immunodeficiency virus (HIV) infection, injection drug use, and the human microbiome is unclear. In this study, we examined the microbiomes of HIV-positive and HIV-negative individuals, both drug-injecting and non-injecting, to identify bacterial community changes in response to HIV and drug use. We utilized a well-established cohort of people who inject drugs in Puerto Rico, a region with historically high levels of injection drug use and an HIV incidence rate disproportionately associated with drug use.

**Methods:**

Using amplicon-based 16S rDNA sequencing, we identified amplicon sequence variants (ASVs) that demonstrated significant variations in the composition of microbial communities based on HIV status and drug use.

**Results and discussion:**

Our findings indicate that the HIV-positive group exhibited a higher abundance of ASVs belonging to the genera *Prevotella*, *Alloprevotella*, *Sutterella*, *Megasphaera*, *Fusobacterium*, and *Mitsuokella*. However, Bifidobacteria and *Lactobacillus* ASVs were more abundant in injectors than in non-injectors. We examined the effect of drug use on the gut microbiome in both HIV-infected and non-infected patients, and found that multiple drug use significantly affected the microbial community composition. Analysis of differential of bacterial taxa revealed an enrichment of *Bifidobacterium* spp.*, Faecalibacterium* spp., and *Lactobacillus* spp. in the multiple drug-injecting group. However, in the non-injecting group, *Parabacteroides* spp., *Prevotella* spp., *Paraprevotella* spp., *Sutterella* spp., *and Lachnoclostridium* spp. The presence of multiple drug-injecting groups was observed to be more prevalent. Our findings provide detailed insight into ASV-level changes in the microbiome in response to HIV and drug use, suggesting that the effect of HIV status and drug injection may have different effects on microbiome composition and in modulating gut bacterial populations.

## 1 Introduction

The prevalence of opioid injection incidence in the United States has increased in numerous previously low-risk regions. In many US states, increases in human immunodeficiency virus (HIV)-1 infections in association with injection drug use have occurred, leading to increased risk for HIV transmission and disease progression (Strathdee and Beyrer, 2015; Peters et al., 2016; Burnett et al., 2018). However, HIV-1 progression occurs at a different rate/pattern among people who inject drugs (PWID) compared to individuals who do not inject drugs. Current biomarkers such as viral load and CD4/CD8 counts have been used to monitor HIV disease progression; however, additional new markers are needed to better understand the interactions between HIV and drug use and disease progression in the PWID population (Moore et al., 2008). Microbiota play a critical role in epithelial barrier function through niche stabilization, production of metabolites, and eliciting mucus formation and synthesis of antimicrobial factors by the gut mucosa (Wells et al., 2017). Therefore, studies of microbiome changes may provide new opportunities to understand interactions between HIV-1 and drug use and to define potential biomarkers associated with dysbiosis.

It is well established that HIV-1 infection and chronic HIV-1 replication in the gut-associated lymphoid tissue (GALT) persist even in individuals with undetectable HIV plasma viral load under long-term treatment inflammation (Deeks et al., 2013; Koay et al., 2018). Changes in the GALT are associated with the disruption of the gut mucosal integrity, subsequently leading to microbial or microbial product translocation. This can lead to an increase in potentially pathogenic bacteria and a decrease in commensal bacteria–dysbiosis (Nowak et al., 2015; Deusch et al., 2018; Zhou et al., 2018). These modifications have demonstrated an increase in *Prevotella* and a decrease in *Bacteroides* in individuals infected with HIV-1 (Gori et al., 2008; Ellis et al., 2011; Pérez-Santiago et al., 2013; Vujkovic-Cvijin et al., 2013; Dillon et al., 2014; Dinh et al., 2015), resulting in chronic systemic inflammation (Dillon et al., 2014; Dinh et al., 2015). These changes are likely due to changes in the immunological balance and shift from anti-inflammatory cytokines to proinflammatory cytokines toward Th1 type responses, enhancing the differentiation of CD4 T cells into Tregs cells, even in spite of antiretroviral treatment (Round et al., 2011; Ishizaka et al., 2021).

Similarly, the utilization of opioids has been associated with modifications in the microbiota (Kolli et al., 2023). In contrast to HIV-1 infection and microbial dysbiosis, little is known about the effects of opioid use on the gut microbiome and the combinatory effect of HIV and drug use on microbiome structure (Hodder et al., 2021; Lloyd et al., 2021). Currently, a limited number of studies have examined the impact of opioid use on the gut microbial community. Studies have shown that opioid use increases intestinal barrier permeability and bacterial species diversity, partly due to an increase in gastrointestinal (GI) transit time. This could be due to enhanced bacterial growth in the colon through opioid use (Vincent et al., 2016; Xu et al., 2017). Metagenomic pathway analyses showed that these changes were associated with enhanced cellular translation, DNA replication, repair, and cell growth and death in the opioid users (Xu et al., 2017). However, it is unknown if these changes also occur in persons with HIV who use opioids. Additionally, little is known about the entire set of interactions between HIV infection, injection drug use, and the human gut microbiome (Satish et al., 2023). Consequently, investigating microbial community dynamics may provide novel opportunities to identify biomarkers in order to better understand the effect of drug use on HIV infection and disease progression.

Here we examined the microbiomes of injecting and non-injecting dug users who are HIV-1 positive and negative in order to identify bacterial community changes in response to HIV-1 and drug use. We used a well-established cohort of injection drug users in Puerto Rico, where there is a historically high level of injection drug use and an HIV incidence rate that is disproportionately associated with drug use. We used Amplicon Sequence Variants (ASVs) to identify sub-OTU level changes in the microbial community composition in order to provide a higher resolution of bacterial composition compared to previous studies (Dillon et al., 2014; Dinh et al., 2015; Armstrong et al., 2018).

## 2 Materials and methods

### 2.1 Ethics statement

This study was approved by the Institutional Review Boards of the University of Nebraska–Lincoln (Lead Institution IRB No. 20190519263FB), Louisiana State University Health Sciences Center, New Orleans, and the Universidad Central del Caribe. Written informed consent was obtained from all study participants, and all study data were deidentified before sample processing, analysis and publication.

### 2.2 Recruitment and sample collection

Recruitment and sample collection were performed as described in Bennett et al. (2023). Briefly, Respondent-driven sampling (RDS) was used to recruit participants from San Juan, Puerto Rico, who fall into the following four 4 groups: (1) drug injectors who are HIV positive, (2) HIV positive and not injecting drugs, (3) drug injectors who are HIV negative, and (4) controls who do not inject drugs and are HIV negative (Hautala et al., 2017; Abadie et al., 2022). After obtaining written informed consent, HIV rapid antibody tests (cat. 90–1019; BioLytical) were performed on each participant to confirm their self-reported HIV status. Additionally, urine samples were collected for rapid drug use tests (CLIA-IDTC-14-BUPa; CLIAwaved, Inc.). Fecal samples were collected at the study site using fecal sample collection containers and Nylon Flocked Swabs (BD) and were frozen at −80°C and was shipped to the University of Nebraska-Lincoln for microbial community analysis on dry ice. Each recruitment group included the following number of participants: (1) drug injectors who are HIV positive (*n* = 15), (2) HIV positive and not injecting drugs (*n* = 55), (3) drug injectors who are HIV negative (*n* = 58), and (4) controls who do not inject drugs and are HIV negative (*n* = 14). Among the HIV positive participants, 62 out of the 70 participants were taking ART. As a result, 88% of the participants were taking ART. Moreover, specific dietary questions were not asked in the questioner except for what a regular meal is for the participant. Based on the responses to this inquiry, the majority of participants eat regular rice, beans, and meat diet.

### 2.3 DNA extraction and sequencing

DNA was extracted from all fecal swabs using the OMEGA Mag-Bind^®^ Soil DNA 96 kit (Omega Bio-tek, Inc., GA, USA), in accordance with the manufacturer’s protocol. In brief, the fecal swab heads were snapped and vortexed in Safe-Lock tubes (Eppendorf, Hamburg, Germany) using 300 μL of sterile 1X PBS to remove the fecal contents from the swab, prior to proceeding with the manufacturer’s protocol. The DNA was extracted using the following modification to the manufacturer’s protocol: (l) following the initial bead-beating at 30 Hz for 10 min, the samples were heated for 10 min at 90^°^C in a water-bath, followed by a second round of bead-beating at 30 Hz for 10 min. Following DNA extractions, the DNA was visualized on a gel to look for degradation and DNA quality and was then used to amplify the V4 hypervariable region of the 16S rRNA gene as described previously (Kozich et al., 2013). Each sample was amplified using uniquely barcoded primers. Each 25L PCR reaction comprised of 1X Terra™ PCR Direct Buffer (Takara Bio Inc., Mountain View, CA, USA), 0.625 units of Terra™ PCR Direct Polymerase (Takara Bio, Inc., Mountain View, CA, USA), 2.5 μM of barcoded primers, and 20–50 ng of fecal DNA. PCR reactions were performed on an Applied Biosystems Veriti 96-well Thermal Cycler (Thermo Fisher Scientific, Inc., Waltham, MA, USA) using the following program: initial denaturation at 98°C for 3 min followed by 25 cycles of denaturation at 98°C for 30 s, annealing at 55°C for 30 s, and elongating at 68°C for 45 s, followed by a final extension of 4 min at 68°C. The resulting PCR products were visualized in a 2% agarose gel (Green BioResearch LLC, Baton Rouge, LA, USA) after gel electrophoresis to confirm the correct product size and to ensure the absence of amplification in negative control samples. Additionally, PCR was performed using known bacterial mock communities (Zymo Research) to identify read thresholds to reduce spurious ASVs that could result from PCR and sequencing artifacts. The resulting barcoded PCR products were normalized using the SequalPrep Normalization Plate Kit (Invitrogen, Carlsbad, CA, USA) according to the manufacturers protocol. Following normalization, equal volumes of normalized amplicons were pooled for further analysis of the prepared libraries and for sequencing. The preparation of PCR plates, amplicon normalization, and pooling were performed with an EpMotion M5073 instrument (Eppendorf, Hamburg, Germany). The quality of the pooled amplicons was then assessed using an Agilent BioAnalyzer 2100 on high sensitivity DNA chips (Agilent, Santa Clara, CA, USA). The library concentration was measured using the DeNovix dsDNA high sensitivity Kit (DeNovix Inc., Wilmington, DE, USA) and was quantified using the Kappa library quantification kit (Roche Biosciences). The resulting dual-index amplicon libraries were sequenced on the Illumina^®^ MiSeq™ platform (Illumina Inc., San Diego, CA, USA) employing the paired-end sequencing strategy (2 × 250 bp) as previously described using V2 kits (Kozich et al., 2013).

### 2.4 Bioinformatic analysis

The bacterial community analysis of the V4 region was performed in R (version 4.1.1) using the phyloseq package (version 1.40.0) (McMurdie and Holmes, 2013). The initial quality filtering steps to remove low quality reads and identification of amplicon sequence variants (ASVs) were performed using the DADA2 pipeline (version 1.24.0) (Callahan et al., 2016). Briefly, potential contaminants originating from reagents were removed using the decontam package (version 1.12.0) Davis et al. (2018). Furthermore, chimeric sequences and any ASVs that were only found in negative controls were removed from the dataset. The negative controls had very few reads (less than 1000 reads). Taxonomy assignment was performed using the DADA2-formatted fasta files obtained from the SILVA Project’s version 138.1 release (Quast et al., 2013). Non-bacterial sequences were subsequently filtered out. Based on the mock communities, a threshold filtering was established, and ASVs with a relative abundance of <0.15% in a given sample and ASVs detected in only one sample were eliminated as previously described (Tom et al., 2024). As a concluding quality control measure, 10 samples comprising <25,000 reads were also removed from the dataset. This resulted in a final quality-filtered ASV table comprising of 168 samples and 806 ASVs, with an average read depth of 97,858 reads/sample. Read counts were normalized to relative abundances to account for differences in sequencing depth. Alpha and beta diversity analyses were performed using the phyloseq package (McMurdie and Holmes, 2013). Alpha diversity was analyzed using the Shannon index (samples were rarefied to an even read depth prior to analysis), while unweighted UniFrac and Bray-Curtis distances were used for beta diversity analyses. Differentially abundant ASVs between the different groups were identified using DESeq2 (Love et al., 2014). All analyses were performed using ASV abundance, and taxonomies were assigned for identified ASVs using the SILVA Project’s version 138.1 release (Quast et al., 2013).

### 2.5 Statistical analysis

Alpha diversity comparison between the different groups was performed using the Mann– Whitney U test. The statistical analysis of beta diversity was performed using Permutational Multivariate Analysis of Variance (PERMANOVA) using the adonis2 function of the R vegan package (Anderson and Walsh, 2013). The significance was determined at alpha = 0.05. A pairwise weighted UniFrac distance matrix (Lozupone et al., 2011) was used to perform principal coordinate analysis (PCoA) to estimate variation between bacterial community composition in the four sample groups using vegan (version 2.6.4) and phyloseq R packages. Differentially abundant ASVs were calculated using the DESeq2 (version 1.36.0) (Love et al., 2014). Differential ASVs were identified using the Benjamini-Hochberg false discovery rate adjusted *p*-value, and *p*-values <0.05 were considered significant. The raw data and pipelines are available on the Fernando Lab github site^1^, where all scripts and data are accessible to the public to replicate the analysis. All 16S sequencing data has been deposited in the Sequence-read archive of the National Center for Biotechnology Information (NCBI) under the following accession number PRJNA1143582.

## 3 Results

### 3.1 Bacterial community composition in HIV and drug injected sampling groups

In order to investigate the effect of HIV status and drug injection on gut microbial community composition, we evaluated the taxonomic distribution and species diversity in each of the four study groups. Following quality filtering, the sequencing data set contained 16,440,230 total reads with an average depth of 97,858 reads per sample. Collectively, 806 ASVs were identified following an abundance and prevalence filtering procedure, based on an abundance filtering of at least 0.15% within a sample and a prevalence filtering of presence in at least two samples to eliminate any spurious ASVs that may have arisen from PCR or sequencing errors as previously described (Tom et al., 2024). This filtering procedure resulted in the retention of 94.2% of the data from the samples studied. The top taxa identified at phylum level in each group is shown in Table 1. The HIV(+)/injecting group was dominated by Actinobacteria, Bacteroidota, Firmicutes, and Synergistota, whereas the HIV(+)/non-injecting group was dominated by Bacteroidota, and Firmicutes. In the HIV(−)/injectors, Actinobacteria, Bacteroidota, and Firmicutes were the most abundant, with HIV(−)/non -injectors dominated by Bacteroidota, and Firmicute. Among the phylum level taxa identified, Actinobacteria abundance was nearly twice as high in drug injecting groups as compared to the non-injecting groups. The phylum Bacteroidota was abundant in all four groups, but there was substantial person-to-person Variation, with the lowest abundance in the control group. Similar to Bacteroidota, Firmicutes abundant in all groups with large person to person variation, but with the highest abundances were identified in the control group. Proteobacteria and Verrucomicrobiota abundances were also highest in the control group. Taxonomic distribution of the bacterial community at level of family and genus is shown in Supplementary Tables S1, S2.

**Table 1:** Phylum-level classification and abundance of ASVs identified.

Phylum	HIV_injecting		HIV_not_injecting		no_HIV_injecting		no_HIV_not_injecting	
	Mean	SD	Mean	SD	Mean	SD	Mean	SD
Actinobacteriota	0.1017	0.1386	0.0579	0.0712	0.1192	0.1255	0.048	0.0467
Bacteroidota	0.4126	0.3111	0.3617	0.2392	0.3183	0.2106	0.2577	0.2236
Campylobacterota	1.00E-04	2.00E-04	0.0012	0.0062	3.00E-04	0.0018	0	1.00E-04
Desulfobacterota	0.004	0.0044	0.0064	0.0102	0.004	0.0051	0.0054	0.0066
Firmicutes	0.44	0.2446	0.5252	0.201	0.5052	0.1806	0.6006	0.2096
Fusobacteriota	9.00E-04	0.0031	0.003	0.0129	1.00E-04	4.00E-04	0	1.00E-04
Proteobacteria	0.0381	0.078	0.0313	0.0641	0.0382	0.0691	0.0529	0.1154
Synergistota	3.00E-04	7.00E-04	0	2.00E-04	0.0014	0.0077	5.00E-04	0.0012
Verrucomicrobiota	0.0024	0.0073	0.0133	0.0465	0.0134	0.0616	0.0349	0.055

**Figure 1:**
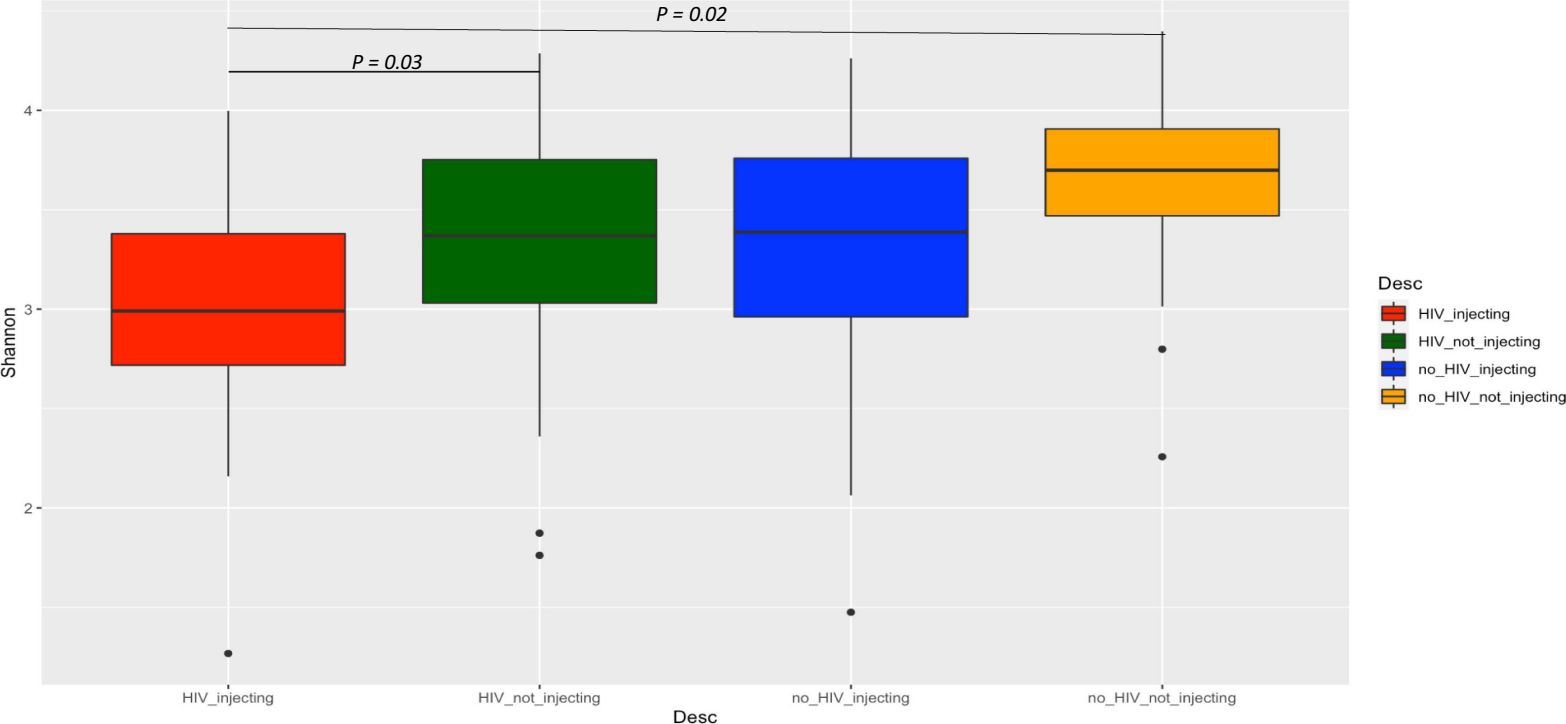
Comparison of alpha diversity among the four groups of subjects based on HIV and injection status.

To further evaluate the impact of HIV status, drug use alone, or in combination on bacterial diversity, we compared the alpha diversity between the four groups using the Shannon index (Figure 1). This analysis indicated that the HIV(+)/injectors had the lowest diversity, while the HIV(−)/non-injector group had the highest diversity. The bacterial diversity within these two groups was significantly distinct (*p* = 0.02). Additionally, the bacterial diversity between the HIV(+)/non-injecting group and the HIV(+)/ injecting group was also significantly different (*p* = 0.03) (Figure 1).

**Figure 2:**
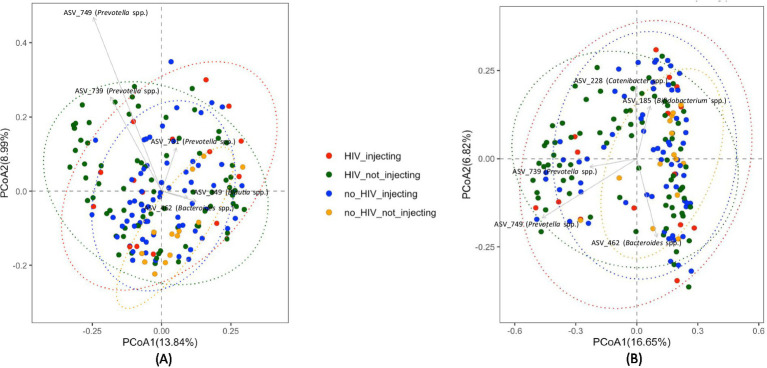
PCoA plots comparing beta diversity among the four groups of subjects based on HIV and injection status **(A)** Unweighted UniFrac distances **(B)** Bray-Curtis distances.

Global differences in bacterial community composition were assessed using Principal Coordinate Analysis (PCoA) and PERMANOVA. This analysis of beta diversity did not reveal clear clustering but identified several taxa that distinguished subject groups (Figure 2). Statistical analysis using the PERMANOVA revealed significant differences between groups (*p* = 0.001). Furthermore, both HIV status (*p* = 0.003) and injection status (*p* = 0.001) were significant factors affecting the structure of the fecal microbiota of the subjects, as indicated by a pairwise analysis of the four groups. This analysis revealed that the bacterial community in the HIV(−)/non-injecting group was significantly different from HIV(+)/non-injecting (*p* = 0.015), and HIV(−)/injecting (*p* = 0.006). Additionally, the bacterial community of the HIV(−)/injecting group was significantly different from the HIV(+)/non-injecting group (*p* = 0.008).

### 3.2 Differentially abundant bacterial ASVs by HIV and drug status

**Figure 3:**
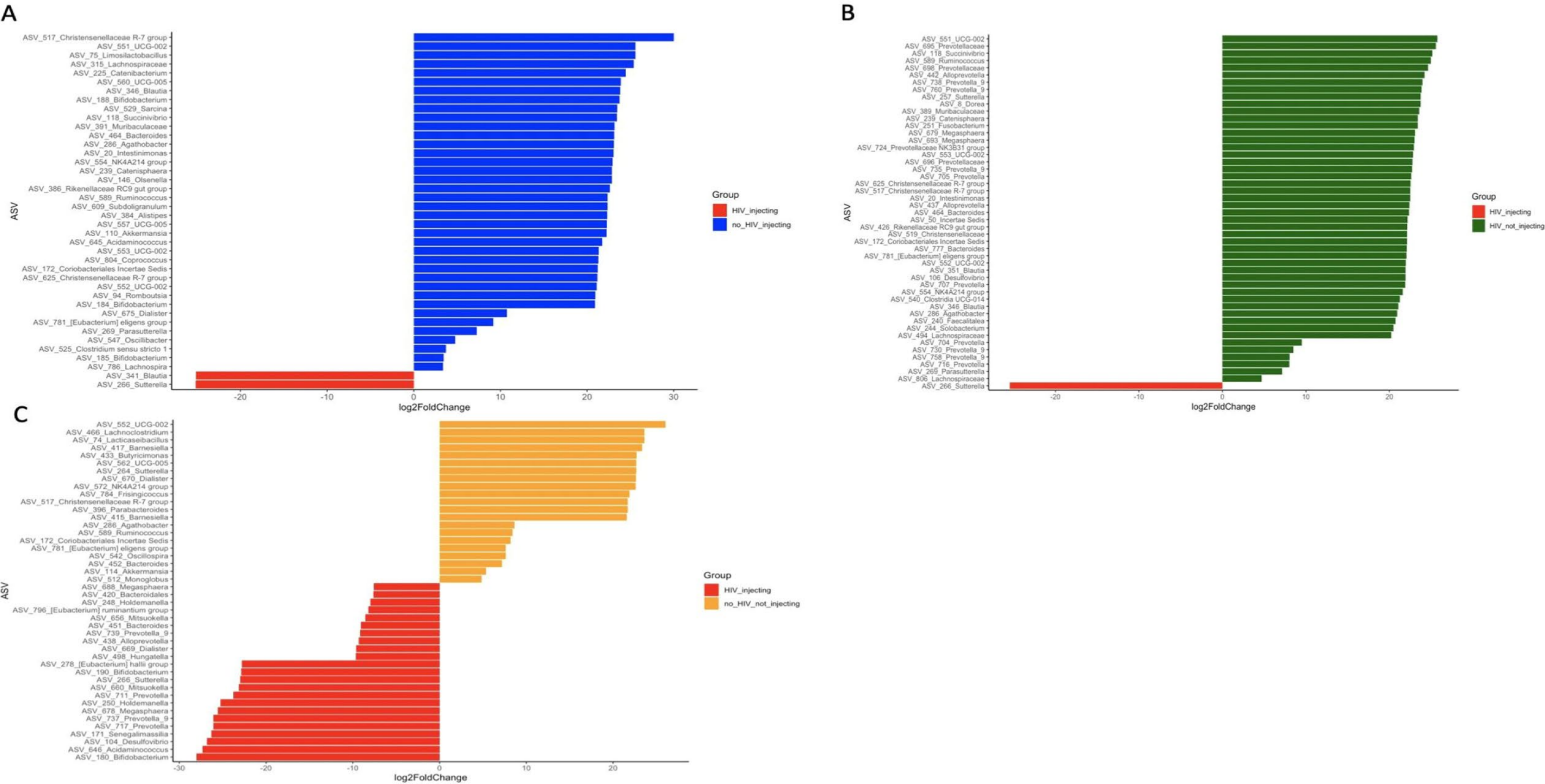
Differentially abundant ASVs between **(A)** HIV(−)/injectors compared to HIV(+)/injectors **(B)** HIV(+)/non-injectors compared to HIV(+)/injectors **(C)** HIV(−)/non-injectors compared to HIV(+)/injectors.

To determine what bacterial taxa are influenced by HIV status and drug injection, we identified differentially abundant ASVs between the HIV(+)/injectors group and the other 3 groups as this group had the lowest diversity. We identified 40, 49, and 44 significantly different ASVs among HIV(−)/injectors, HIV(+)/non-injectors, and HIV(−)/non-injectors, respectively (Figure 3 and Supplementary Tables S3–S5).

**Figure 4:**
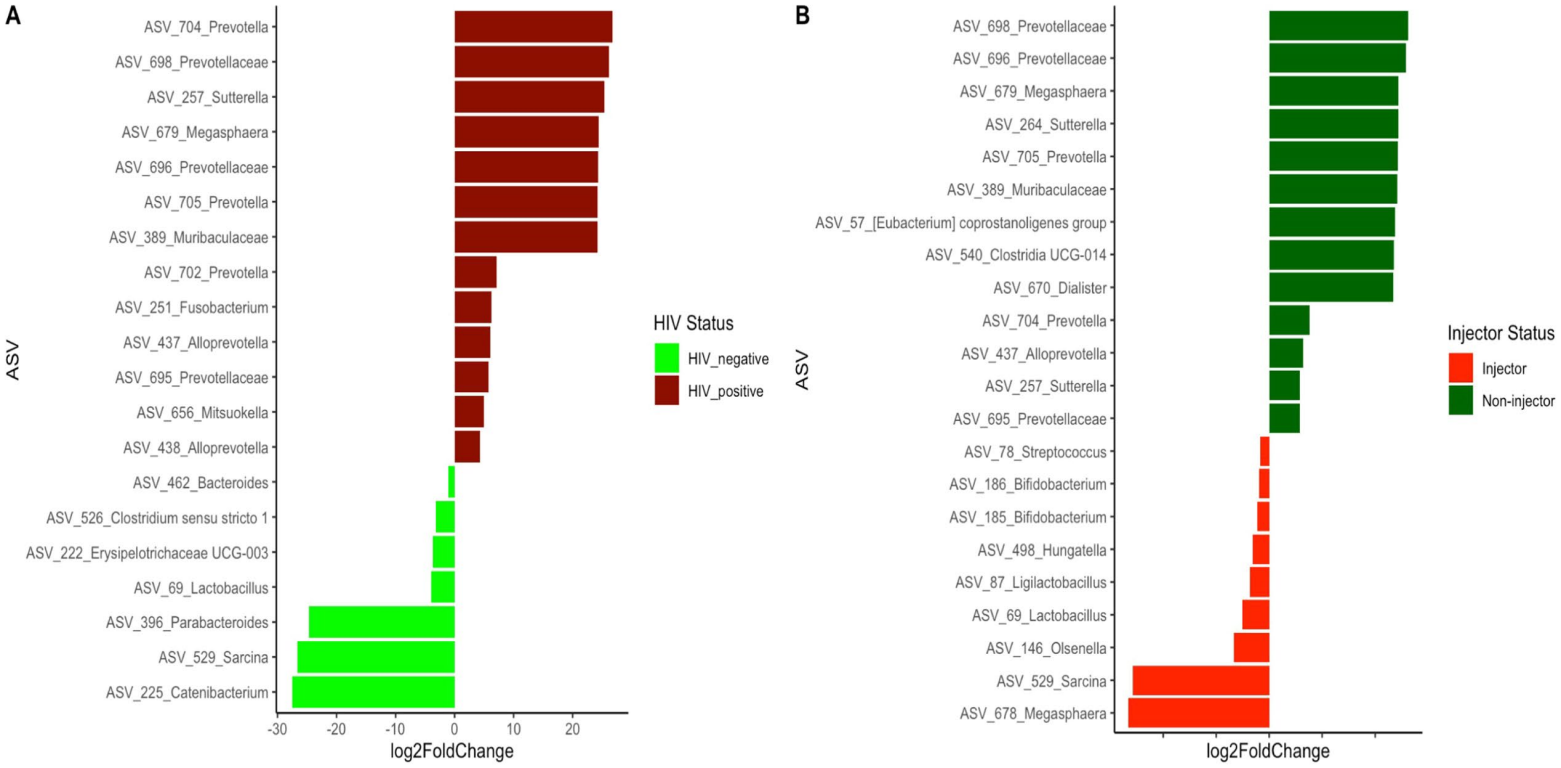
Differentially abundant ASVs between **(A)** HIV(+) subjects compared to HIV(−) subjects **(B)** drug non-injectors compared to drug injectors.

Since both HIV status and injection status were identified as significant factors affecting the bacterial community structure of the subjects, we further examined the differentially abundant ASVs present between the HIV(+) and HIV(−) groups, as well as between the injector groups (Figure 4 and Supplementary Table S6). A total of 20 differentially abundant ASVs were identified based on HIV status, and 22 differentially abundant ASVs were identified based on injector status (Supplementary Table S7). It is noteworthy that 11 ASVs were prevalent among the differentially identified ASVs based on HIV status and injector status.

### 3.3 Identifying core ASVs in the HIV(−)/non-injector group and their representation in the other groups

**Figure 5:**
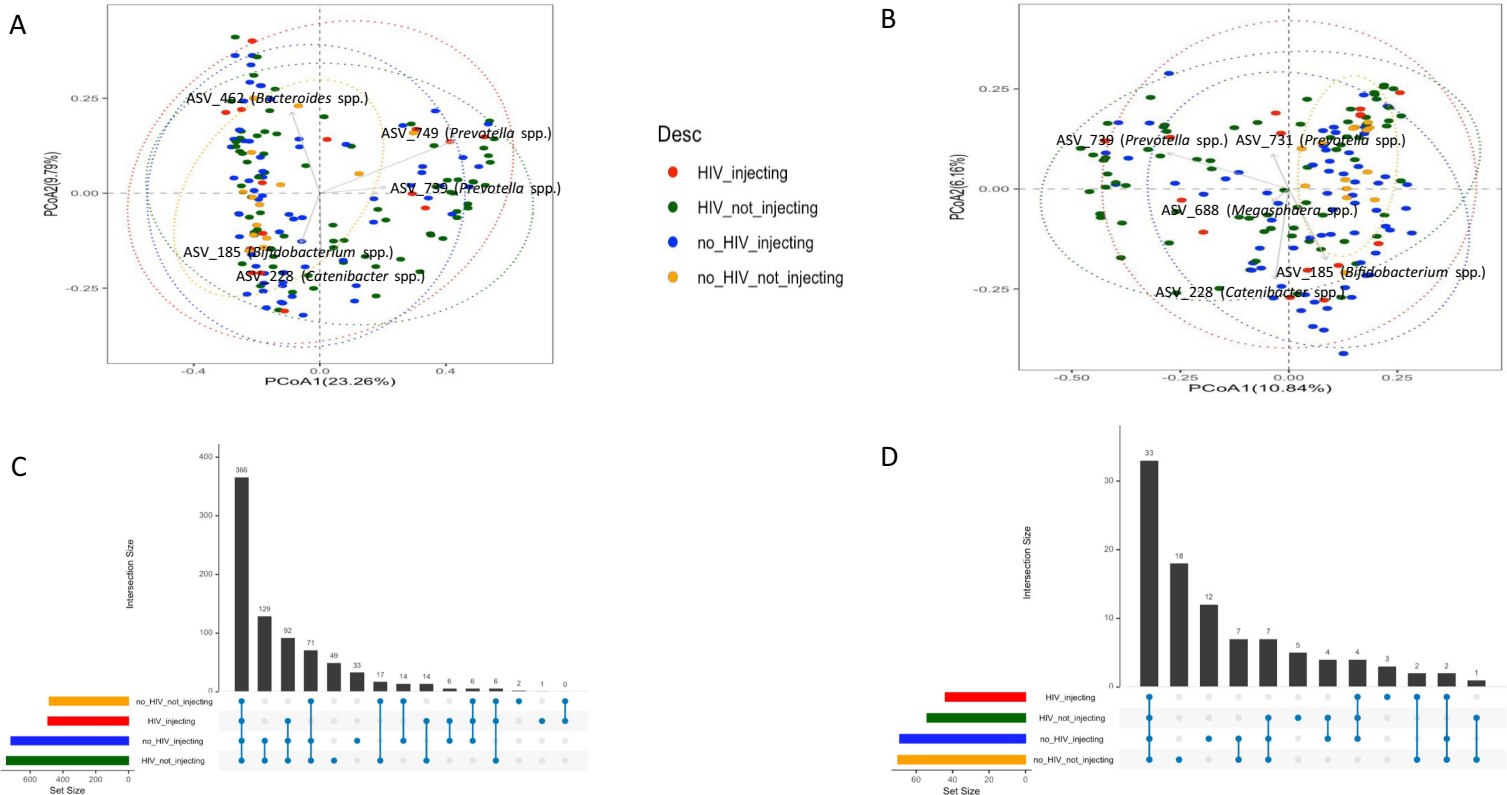
PCoA plots (Bray-Curtis distances) comparing beta diversity for the **(A)** core ASVs among the four groups of subjects based on HIV and injection status **(B)** beta diversity among the four groups of subjects after removing the 37 common core ASVs. UpSet plots depict **(C)** the number of shared ASVs across the different groups **(D)** number of shared core ASVs across the different groups.

We considered the subjects without HIV or injected drug use to be (HIV(−)/non-injectors group) as ‘healthy’ subjects and defined a core set of bacterial taxa that are represented in that population from San Juan, Puerto Rico. A ‘core’ microbiota was defined for all groups by identifying ASVs that were present in at least 50% of fecal samples collected from within each group, with an abundance of 0.01% as previously described (Kellogg, 2019). This analysis revealed that 44 ASVs that accounted for 51.60% of the reads in the HIV(+)/injecting group, 54 ASVs that accounted for 50.4% reads in the HIV(+)/non-injecting groups, 69 ASVs that accounted for 61.5% of the reads in the HIV(−)/injecting group and 70 ASVs that accounted for 65.1% of the reads in the HIV(−)/non-injecting group. The taxonomic information for each group is present in Supplementary Tables S8–S11. A set of 33 ASVs were identified as common across the 4 groups and accounted for 39.0% of the total reads (Supplementary Table S12). Interestingly, the core bacteria found in all 4 groups contained bacterial species that are considered to be beneficial to human health, such as *Faecalibacterium* spp., *Blautia* spp., and *Bifidobacterium* spp. After removing the core bacterial ASVs found in all sampling groups, we conducted Principal Coordinate Analysis (PCA) and PERMANOVA analysis to determine whether the remaining core ASVs are capable to distinguishing each sampling group. The PCoA analysis with core taxa and after removing the 33 common core ASVs is shown in Figures 5A,B (Supplementary Figure S1 shows the same PCoA plots based on Unweighted UniFrac distances) with an ASV distribution in Figures 5C,D. Although PCoA did not distinguish groups, PERMANOVA analysis identified the core taxa that could significantly distinguish each group (Unweighted UniFrac *p* = 0.001, and Bray-Curtis *p* = 0.035). After removal of the common ASVs, the HIV(+) injecting core contained 11 ASVs, as shown in Supplementary Table S13. The HIV(−) injecting core contained 36 core ASVs after removal of common core ASVs, as shown in Supplementary Table S14. The HIV(+) non-injecting core included 21 ASVs, which are shown in Supplementary Table S15. The control group that contained HIV(−) non-injecting participants contained the largest amount of core ASVs, which included 37 ASVs. These are shown in Supplementary Table S16. The core taxa belonging to each group before and after common core ASV removal are shown in Supplementary Tables S8–S11, S13–S16, respectively.

### 3.4 Microbiome structure based on drug usage

**Figure 6:**
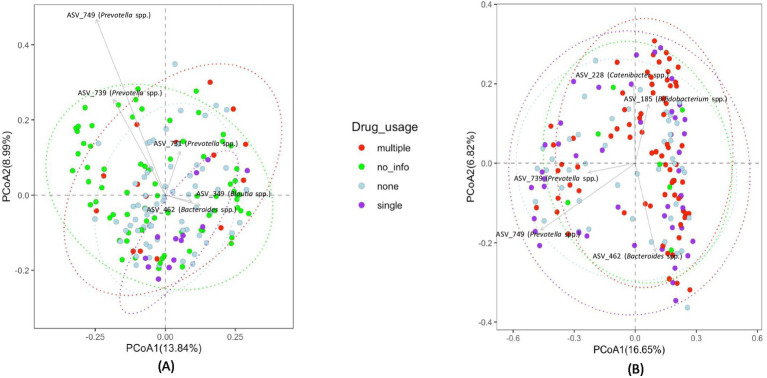
PCoA plots depicting clustering of fecal bacterial communities of subjects based on drug usage **(A)** Unweighted UniFrac distances **(B)** Bray-Curtis distances.

**Figure 7:**
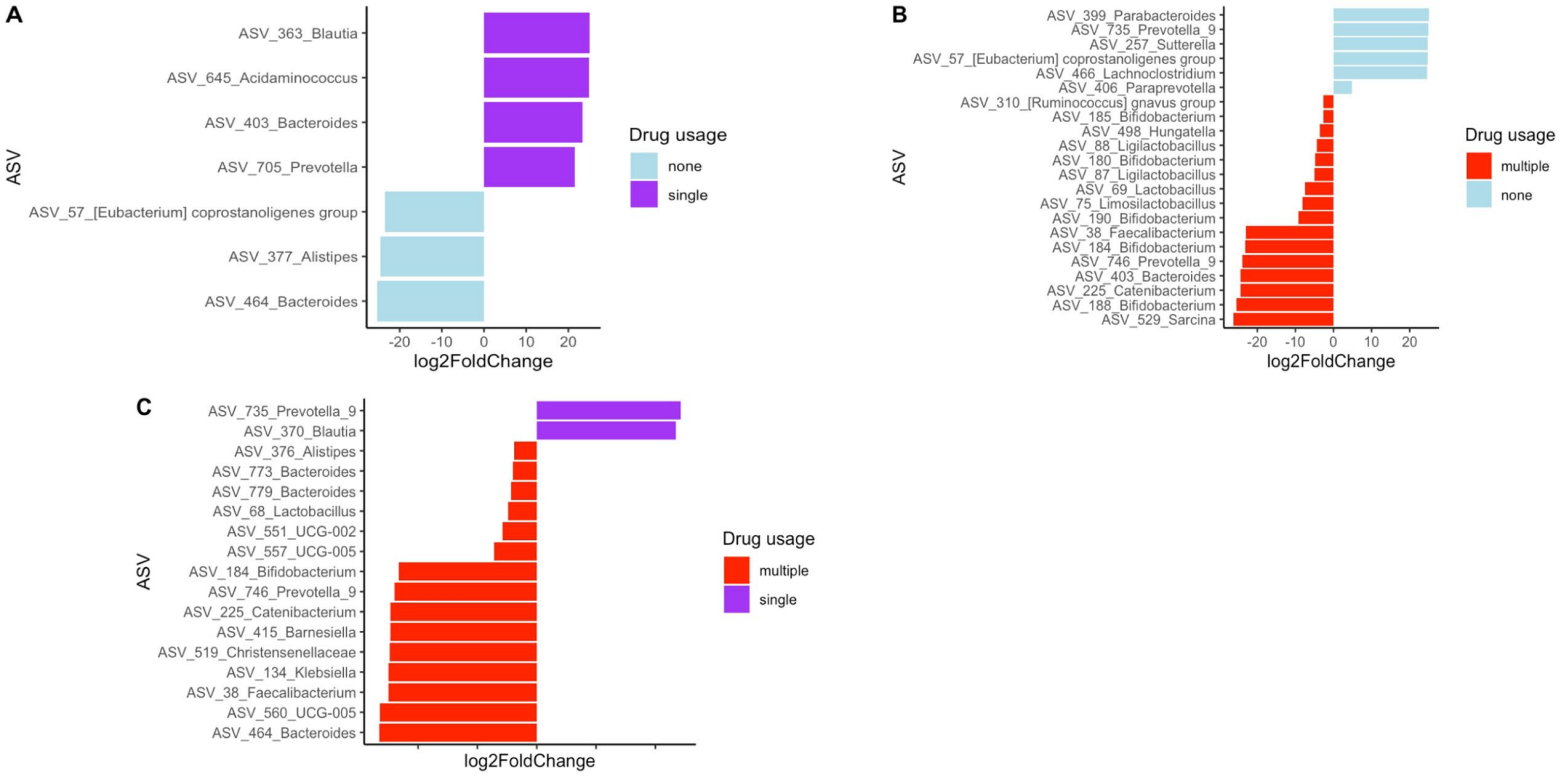
Differentially abundant ASVs between **(A)** single drug users compared to no drug users **(B)** no drug users compared to multiple drug users **(C)** single drug users compared to multiple drug users.

The sample subjects reported using various types of drugs. Therefore, we attempted to assess how drug level and usage impacted gut bacterial community composition. However, the diversity of drugs used by the study participants was too large to categorize by drug type, with a very small sample size for each drug or drug combination. Therefore, we categorized drug usage among subjects as the number of different drugs used as follows: 0 drugs—none, 1 drug—single, or 2 or more than 2 drugs—multiple. The clustering of fecal samples based on these categories is presented in Figure 6. Statistical analysis using PERMANOVA revealed that the number of drugs used had a significant effect on bacterial community composition (*p* = 0.017 for unweighted UniFrac and *p* = 0.044 for Bray-Curtis matrices). Additionally, differential abundance analysis of bacterial taxa was performed to identify enrichment or depletion of bacterial taxa due to the use of single drugs/no drug usage and multiple drugs. This analysis identified 22 ASVs to be significantly different between non-users and users of multiple drugs, and is listed in Supplementary Table S17. The comparison of multiple drug injectors to single drug injectors is shown in Supplementary Table S18. This analysis identified 17 ASVs that were differentially abundant between the two groups. When comparing non-drug users to single drug users, 7 ASVs were identified as different between the groups, as shown in Supplementary Table S19. Figure 7 shows all the differential ASVs identified among the various drug usage groups.

## 4 Discussion

HIV-1 progression among people who inject drugs (PWID) occurs at a different rate/pattern compared to individuals who do not inject drugs. Current biomarkers, such as viral load and CD4 counts, may not provide a clear understanding of disease progression or severity. There is little known about the full extent of interactions between HIV infection, injection drug use, and the human microbiome. Here, utilizing a well-established cohort of injection drug users in Puerto Rico, we examined bacterial communities of drug injecting and non-injecting individuals who were HIV positive and negative in order to identify bacterial community changes in response to HIV and drug use.

### 4.1 Bacterial community composition is influenced by HIV and drug status

Previous studies investigating the microbiome in HIV patients and HIV-negative controls have demonstrated inconsistent results attributable to a small sample size, inappropriate controls, and regional and dietary differences (Williams et al., 2016). We conducted very deep sequencing of the fecal microbiome (5X-10X) on a sample size of 168 with additional mock community-based filtering approaches to investigate bacterial communities of HIV- positive and negative individuals that inject or do not inject drugs. Our analysis of the HIV(−)/non-injecting and HIV(+)/non-injecting groups is concordant with previous studies that reported that HIV infection to have no effect on bacterial alpha diversity compared to HIV(−) controls (Dinh et al., 2015). However, our data suggest injection drug use may result in dysbiosis and decreased bacterial alpha diversity. This notion is further supported by the observation that the HIV(+)/non-injecting group exhibited a significantly higher alpha diversity (*p* = 0.03) in comparison to the HIV(+)/injecting group. Our data suggest that HIV alone or drug injection alone does not alter bacterial alpha diversity; however, HIV and drug injection in combination leads to a significant decrease in alpha diversity. In order to further investigate how bacterial community composition is influenced by HIV infection and drug use, we performed beta diversity analysis across the four study groups. While principal coordinate analysis using either the Bray-Curtis or Unweighted UniFrac matrix did not show clear clustering of sampling groups, PERMANOVA analysis identified significant differences in the bacterial community based on HIV status (*p* = 0.003) and drug injection (*p* = 0.001). The major drivers influencing bacterial community structuring included multiple *Prevotella* spp. and *Bacteroides* spp. Similar to our findings, previous studies have reported an increased abundance of *Prevotella* in HIV-infected adults compared to controls (Ling et al., 2016; Gootenberg et al., 2017). However, these studies did not investigate drug injection effects on microbial community compositional changes. Here, we demonstrate that drug injection in HIV-positive individuals leads to decreased diversity and significant microbiome compositional changes.

### 4.2 Bacterial biomarkers that are influenced by HIV status and drug usage

In order to identify the bacterial genera or ASVs significantly altered by HIV infection and drug use, we identified differentially abundant ASVs between each of the four sampling groups. Out of the 40 differentially abundant ASVs identified between HIV(+)/injectors and HIV(−)/injectors, several ASVs belong to *Bifidobacterium* spp., *Akkermansia* spp., and *Alistipes* spp. were identified as significantly more abundant in the HIV(−)/injector group (Supplementary Table S3 and Figure 3A). *Bifidobacteria* spp. have been described as members of a healthy gut and to have positive health benefits including resistance against pathogen colonization through competitive exclusion, modulation of the immune system, and nutrient digestion (Roberfroid et al., 1995; Hooper et al., 2002; O’Hara and Shanahan, 2007; O'Callaghan and van Sinderen, 2016). As such, a decrease in the number of *Bifidobacteria* spp. in the HIV(+)/injector group would suggest increased risk of pathogen colonization and disease susceptibility. Additionally, *Akkermansia* spp. abundance was lower in the HIV(+)/injecting group compared to the HIV(−)/injecting group. *Akkermansia* spp. has been identified as a mucin-degrading organism that helps maintain a healthy gut barrier and mucus layer thickness, leading to immune modulation and reduced inflammation (Cani et al., 2022). Lower abundance of *Akkermansia* spp. has been observed in multiple diseases including obesity, diabetes, liver steatosis, and inflammation (Cani et al., 2022). The reduced abundance of *Akkermansia* spp. in the HIV(+)/injecting group may suggest that drug injection exacerbates microbiome alterations that potentiate the gut permeability. As such, the microbial translocation previously reported may be a result of changes in *Akkermansia* spp. that contribute to the chronic inflammation observed during HIV infection (Nowak et al., 2015; Deusch et al., 2018; Zhou et al., 2018). *Alistipes* spp. were also found to be low in the HIV(+)/injecting group. These bacteria have bile acid talerance (David et al., 2014; Gootenberg et al., 2017) with anti-inflammatory properties and are suggested to provide protection against colitis (Parker et al., 2020). As such, decreased *Alistipes* spp. may suggest dysbiosis in HIV(+)/injectors leading to gut inflammation and pathogen colonization. Only two bacterial species were found to be enriched in the HIV(+)/injecting group compared to the HIV(−)/injecting group Sutterella spp., Blautia spp., and *Sutterella* spp. has been described as a pathogen that causes gastrointestinal disease (Mukhopadhya et al., 2011; Hiippala et al., 2016). This further supports our observation that drug use may increase the risk of pathogen colonization and translocation in HIV patients with an imbalanced bacterial community structure. *Blautia* spp. is a newly described species from the human gut, for which its role in the human microbiome is unclear. Our results suggest that *Blautia* spp. is a bacterial species that is associated with drug injection in the context of HIV infection.

A total of 49 ASVs were identified to total of 49 ASVs were identified exhibit a differentially high abundance between the HIV(+)/injecting group and the HIV(+)/non-injectors, thereby enabling the identification of the impact of drug injection on the microbiome in HIV(+) adults (Figure 3B and Supplementary Table S4). This analysis identified multiple ASVs belonging to the genus *Prevotella* and the families *Oscillospiraceae* and *Christensenellaceae* to be greater in HIV(+)/non-injectors (Figure 3B and Supplementary Table S4). Previous studies have reported that *Prevotella* has increased abundance in HIV-infected adults compared to controls (Ling et al., 2016; Gootenberg et al., 2017). However, we observed that drug injections led to a decrease in *Prevotella* compared to non-injectors suggesting that the microbiome responded differently to the injection drugs compared to HIV status. Additionally, the occurrence of *Christensenellaceae* was decreased in response to drug injection. It has been reported that *Christensenellaceae* exhibit a decrease in inflammatory bowel disease (IBD) (Mancabelli et al., 2017), Crohn’s disease (Pascal et al., 2017), and irritable bowel syndrome (IBS) (Jalanka-Tuovinen et al., 2014) indicating that the decrease in abundance of this family is associated with inflammation (Waters and Ley, 2019). As such, the decrease in *Christensenellaceae* in the injecting group suggests that drug injection may lead to a dysbiosis in the gut, leading to gut inflammation. We also observed a decrease in the number of ASVs belonging to the family *Oscillospiraceae* during drug injection. *Oscillospiraceae* are known to produce butyrate (Yang et al., 2021), which has been shown to reduce mucosal inflammation and improve the epithelial defense barrier (Canani et al., 2011). Therefore, the decrease in *Oscillospiraceae* abundance associated with drug injection suggests that drug injection may result in decreased gut barrier function, thereby causing leaky gut. Similar to the comparison between HIV(+)/injecting and HIV(−)/injecting group, the only bacterial species enriched in the HIV(+)/injecting group was *Sutterella* spp.

Among the groups that were HIV(+)/injecting and HIV(−)/non-injecting, 44 significantly differentially abundant ASVs were identified (Figure 3C and Supplementary Table S5). A different ASV belonging to *Akkermansia* spp. was enriched in the HIV(−) non-injecting group compared to the HIV(+)/injecting group. Additionally, two distinct ASVs belonging to *Bifidobacterium* spp. displayed increased abundance in the HIV(+)/injecting group. Since our ASV-based read binning is based on a single nucleotide difference in a given location in the 16S rRNA gene, we are identifying sub-OTU level variation in the bacterial community. As such, sub-OTU level variation within different bacteria belonging to the same higher taxonomic rank may contribute to HIV status and drug injection differently. Therefore, this study provides microbial community differences at a higher resolution than reported previously (Dillon et al., 2014; Dinh et al., 2015; Armstrong et al., 2018).

A total of 20 ASVs were identified to exhibit significant differences based on HIV status (Supplementary Table S6), wherein the HIV(+) group exhibited a higher abundance of ASVs belonging to the genus *Prevotella* and Family *Prevotellaceae*. This observation is consistent with previous reports where HIV(+) patients are shown to have an increase in this taxa (Ling et al., 2016; Gootenberg et al., 2017) and have been linked to pro-inflammatory properties (Armstrong et al., 2018). Additionally, several ASVs belonging to the genera *Alloprevotella*, *Sutterella*, *Megasphaera*, *Fusobacterium*, and *Mitsuokella* were found to be high in abundance in HIV(+) subjects. A study examining the vaginal bacterial community associated with the risk of HIV acquisition identified *Megasphaera* spp. abundance to be significantly associated with HIV acquisition (McClelland et al., 2018). Although not previously reported, we believe that our deep sequencing may have led to the discovery of this important bacterial marker for HIV status. Similar to our observations, previous studies have also reported an increase in the abundance of *Fusobacterium* in individuals with HIV (Lee et al., 2018). An increase in the genus *Mitsuokella* in HIV(+) patients has been reported previously (Lozupone et al., 2013). Therefore, our observations of an increased abundance of *Fusobacterium* and *Mitsuokella* are consistent with previous reports.

Numerous genera, including *Bacteroides*, *Lactobacillus*, *Parabacteroides*, *Clostridium sensu stricto1*, *Erysipelotrichaceae UCG-003*, *Sarcina*, and *Catenibacterium*, were identified to have a significantly higher abundance in HIV(−) participants. Previous studies comparing HIV status have indicated that *Bacteroides* and *Parabacteroides* genera are prevalent in individuals with high HIV (−) (Lozupone et al., 2013). However, the study by Lozupone et al. did not describe what species may be associated with HIV(−) individuals. It is suggested that *Bacteroides* spp. and *Parabacteroides* spp. may be associated with HIV(−) individuals. Studies have reported *Lactobacillus* abundance changes in HIV patients (Gori et al., 2008). Furthermore, studies have reported that *Lactobacillus* has a CD4 receptor on the cell surface, and that *Lactobacillus* can use this receptor to bind to HIV and reduce HIV infection (Su et al., 2013). Therefore, the depleted *Lactobacillus* abundance in HIV(+) participants compared to HIV(−) participants may contribute to active HIV infections in HIV(+) individuals. Furthermore, *Erysipelotrichaceae UCG-003* and *Catenibacterium* abundance decreases have been reported previously in HIV(+) individuals (Villoslada-Blanco et al., 2022) and are consistent with our observations.

Differential ASVs based on drug injection status identified *Bifidobacterium* and *Lactobacillus* ASVs to be higher in abundance in injectors compared to non-injectors. This was surprising, as we expected drug use to further affect bacterial community composition. This analysis was performed irrespective of HIV status and may suggest that the effect of drug injection alone changes the gut microbial community differently than HIV status. However, a study by Barengolts et al. reported a decrease in Bifidobacteria during opioid use, which supports our observation (Barengolts et al., 2018). *Prevotella* abundance was high in non-injectors compared to injectors and is consistent with previous studies (Ling et al., 2016; Gootenberg et al., 2017). It is interesting that 11 ASVs were common among the differentially identified ASVs based on HIV status and injector status. ASVs belonging to *Prevotella*, *Megasphaera hexanica*, *Alloprevotella* and *Sutterella* were identified as common among HIV(+) and non-injectors, while those belonging to the genera *Lactobacillus* and *Sarcina* were identified as common among HIV(−) and injecting groups. Here, we describe ASV-level changes in the bacterial composition that are impacted by HIV status and drug injection, and provide more resolution on microbiome species variation than reported in previous studies (Dillon et al., 2014; Dinh et al., 2015; Armstrong et al., 2018).

### 4.3 Core ASVs in the HIV(−)/non-injector group and their representation in the other groups

Considering subjects who are HIV(−) /non-injecting as being ‘healthy’ subjects, and identified a core set of bacterial taxa that represent the healthy study population in San Juan, Puerto Rico. According to methods, ASVs were detected in at least 50% of the fecal samples collected from this group, with an abundance of 0.01% as previously described (Kellogg, 2019). We hold the belief that the low reads represented in the core (51–66%) is due to the large person to person variation in the study population. We have identified the fundamental bacterial community for each group and eliminated the shared ASVs that are common to all four sampling groups. A set of 33 ASVs were identified to be common across the 4 groups, accounting for 39% of the total reads (Supplementary Table S12). These 33 ASVs could be considered as the autochthonous bacterial population found within the sampled population. Interestingly, the core bacteria found in all four groups contained bacterial species that are considered to be beneficial to human health, such as *Faecalibacterium* spp., *Blautia* spp., *Roseburia* spp., *Bacteroides* spp., and *Bifidobacterium* spp. To identify presumptive bacterial biomarkers that describe each sampling group, the 33 common core taxa were removed and analyzed to evaluate if the remaining core taxa could be used to describe unique microbes that are enriched within each sampling group to use as potential biomarkers.

After removal of the common ASVs, the HIV(+)/injecting core contained multiple ASVs belonging to the genus *Bacteroides*, *Desulfovibrio* spp., *Streptococcus* spp., and *Roseburia* spp. (Supplementary Table S13). The HIV(−)/injecting core contained, *Faecalibacterium* spp., *Blautia* spp., *Lachnoclostridium* spp., and *Bacteroides* spp. (Supplementary Table S14). The HIV(+)/non-injecting core included ASVs belonging to *Fecalibacterium* spp., *Lachnoclostridium* spp., and *Bifidobacterium* spp. (Supplementary Table S15). The control group that contained HIV(−)/non-injecting participants contained the largest amount of core ASVs, which included *Alistipes* spp., *Faecalibacterium* spp., *Bacteroides* spp., *Akkermansia* spp., *Lachnoclostridium* spp., and *Lachnospiraceae* spp. (Supplementary Table S16). The higher resolution obtained using ASV binning makes this study unique from previous studies describing the effect of microbiome changes based on HIV status. As such, further investigation of the differential ASVs shows that strain level differences in the microbiome are important in identifying the effects of HIV status and drug injection on the microbiome and how microbiome changes would contribute to disease susceptibility. The core ASVs identified here provides important new information into how different strains within the same species may respond based on HIV and drug use. Therefore, we believe that the “unique” core organisms identified in this study may act as valuable indicator species of HIV status and injectable drug use.

### 4.4 Influence of drug injection on bacterial community composition

Although studies have examined changes in gut bacterial community composition with HIV status, studies investigating HIV status together with drug use are limited. Here we attempted to identify the effect of drug use on the gut microbiome in HIV infected and non-infected patients. Due to the large variability of drugs used, it was difficult to characterize the study population based on drug type, so we analyzed the data based on drug use or not and multiple vs. single drug use. Although clear clustering in the principal coordinate analysis was not present (Figure 4), statistical analysis using PERMANOVA revealed that the use of multiple drugs had a significant effect on the microbial community composition (*p* = 0.017 for unweighted UniFrac and *p* = 0.044 for Bray-Curtis matrices). Additionally, differential analysis of bacterial taxa was performed to identify enrichment or depletion of bacterial taxa due to the use of drugs. Twenty-two ASVs were identified to be different between non-users and participants using multiple drugs (Supplementary Table S17). A majority of the bacterial taxa identified as differential were enriched in the group using multiple drugs. This included an increased abundance of *Bifidobacterium* spp., *Faecalibacterium* spp., and *Lactobacillus* spp. In the non-injecting group *Parabacteroides* spp., *Prevotella* spp., *Paraprevotella* spp., *Sutterella* spp. and *Lachnoclostridium* spp. were more abundant compared to the multiple drug-injecting group. When comparing multiple drug injectors to single drug injectors (Supplementary Table S18), only 2 ASVs had a higher abundance in the single drug group (*Blautia* spp. and *Prevotella* spp.). Fifteen ASVs were identified to have higher abundance in the multiple drug injection group (*Alistipes* spp., *Bacteroides* spp., *Lactobacillus* spp., *Bifidobacterium* spp., *Barnesiella* spp., and *Feacalibacterium* spp.). When comparing non-drug users to single drug users (Supplementary Table S19), *Alistipes* spp. and *Bacteroides* spp. was significantly higher in the non-injecting group, whereas *Blautia* spp., *Acidaminococcus* spp., *Bacteroides* spp., and *Prevotella* spp. was higher in the single drug–injected group. It was surprising to see that the multiple drug injecting group was enriched for organisms such as *Alistipes* spp., *Bacteroides* spp., *Lactobacillus* spp., *Bifidobacterium* spp., and *Feacalibacterium* spp. as these organisms are considered members of a healthy gut. It is plausible that the effects of HIV status and drug injection may work against each other in modulating gut microbial populations. This is seen in the reduction of *Prevotella* species during drug use, whereas an increase in *Prevotella* is reported in HIV patients. Additionally, in this study, we are evaluating the gut microbiome at a very high resolution compared to previous studies using ASVs. As such, we are identifying sub-OTU level differences in microbial taxa where some bacteria of the same species are selected in the multiple drug injecting group, whereas the non-injecting group may possess distinct bacterial species belonging to the same taxonomic rank. However, further analysis using shotgun metagenomics or other high-resolution techniques would be necessary to confirm strain-specific or functional differences within the bacterial community. Additionally, one of the biggest challenges in this study was recruitment. As a result, there is a large disparity in the number of participants within each group. Therefore, further studies are needed to provide more detailed insight into the strain level changes in the microbiome in response to HIV and drug use.

## 5 Conclusion

We examined the microbiomes of HIV- positive and HIV-negative individuals, both drug-injecting and non-injecting, to identify bacterial community changes in response to HIV and drug use. Utilizing amplicon sequence variants (ASVs) of the 16S rRNA gene, we identified significant differences in microbial community composition based on HIV status and drug use. When examining the effect of drug use on the gut microbiome in both HIV-infected and non-infected patients, we found that multiple drug uses significantly affected the microbial community composition. Our findings provide detailed insights into ASV-level changes in the microbiome in response to HIV and drug use, suggesting that the effects of HIV status and drug injection may have different effects on microbiome composition and in modulating gut bacterial populations.

## Data Availability

All 16S sequencing data has been deposited in the National Center for Biotechnology Information (NCBI) sequence read archive under the following accession number PRJNA1143582.

## Footnotes

^1^ https://github.com/FernandoLab/

## Supplementary material

SUPPLEMENTARY FIGURE S1 PCoA plots (Unweighted UniFrac distances) comparing beta diversity for the **(A)** core ASVs among the four groups of subjects based on HIV and injection status **(B)** beta diversity among the four groups of subjects after removing the 33 common core ASVs’.
