# Supplementary figures and images for: Gut microbiota profiling in injection drug users with and without HIV-1 infection in Puerto Rico
Source: Front Microbiol. 2024 Nov 26;15:1470037. doi: 10.3389/fmicb.2024.1470037 (PMC11652967; doi:10.3389/fmicb.2024.1470037)

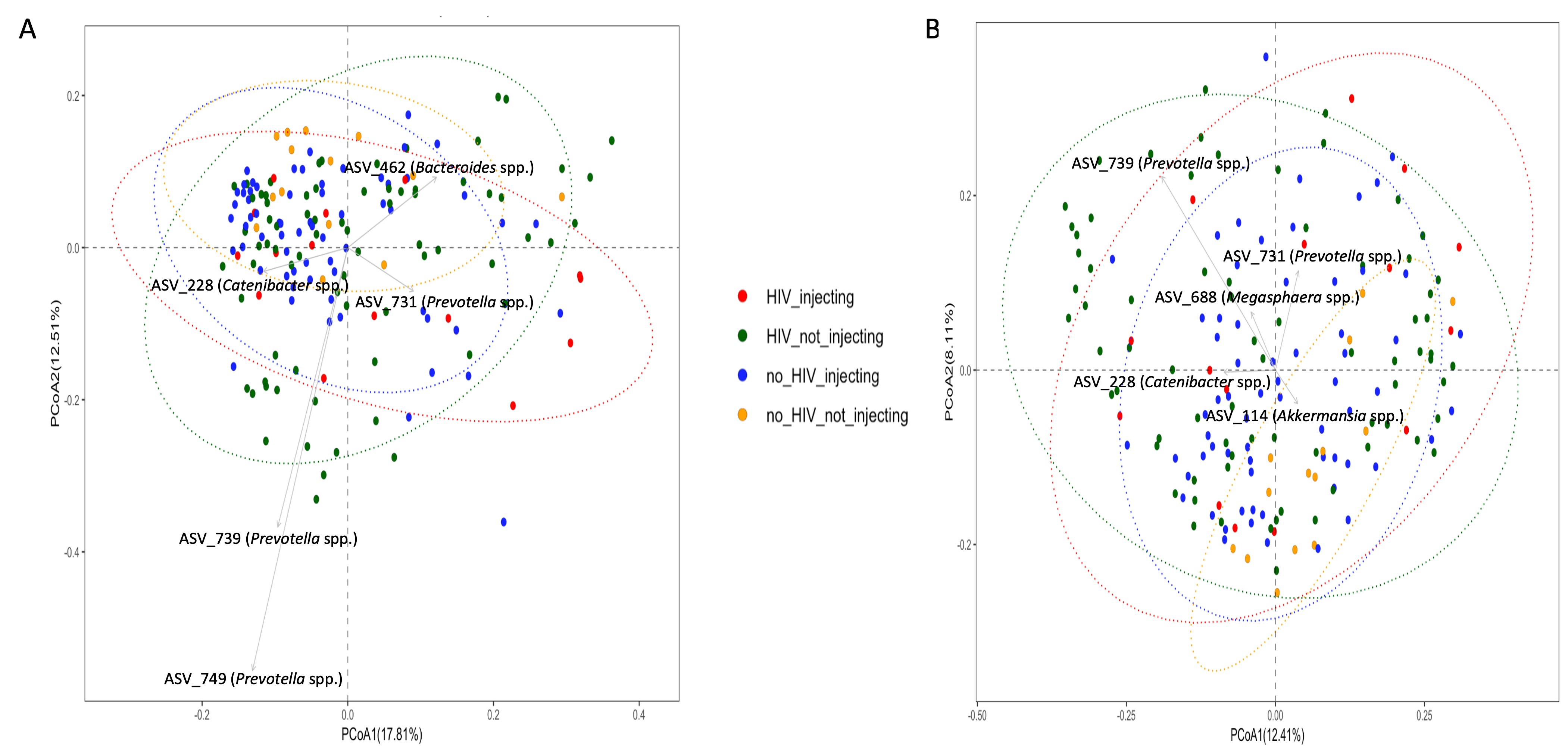

Supplement: SUPPLEMENTARY FIGURE S1 — PCoA plots (Unweighted UniFrac distances) comparing beta diversity for the (A) core ASVs among the four groups of subjects based on HIV and injection status (B) beta diversity among the four groups of subjects after removing the 33 common core ASVs’. [file Image_1.jpg]
